# Abdominal Obesity among Outpatients in a Tertiary Level Eye ENT Hospital: A Descriptive Cross-sectional Study

**DOI:** 10.31729/jnma.7253

**Published:** 2022-01-31

**Authors:** Madan Prasad Upadhyay, Sanjib Kumar Upadhyay, Janak Raj Bhattarai, Bijay Khatri, Rajan Shrestha

**Affiliations:** 1 BP Eye Foundation, Hospital for Children Eye ENT and Rehabilitation Services, Bhaktapur, Nepal; 2 Academic and Research Department, BP Eye Foundation, Hospital for Children, Eye, ENT and Rehabilitation Services, Bhaktapur, Nepal

**Keywords:** *hospital*, *Nepal*, *obesity*, *outpatient*, *screening*

## Abstract

**Introduction:**

Abdominal obesity, as abnormal fat accumulation that presents a risk to health, is a global epidemic. There is evidence to support a trait of abdominal adipose deposition despite normal body mass index in south asian populations with greater cardiometabolic risks. Thus, this study aimed to find out the prevalence of abdominal obesity using the waist to height ratio among outpatients in a tertiary level hospital.

**Methods:**

This descriptive cross-sectional study was conducted among outpatients at a tertiary level hospital in Nepal from January 2016 to December 2018. Ethical approval was taken from the Ethical Review Board of Nepal Health Research Council (Reference no. 207/2019). Convenience sampling was done. The data were entered into excel and analyzed using Statistical Package for Social Sciences version 24. Point estimate at 99% confidence interval was calculated along with frequency and proportion for binary data.

**Results:**

Among 25,511 participants, 21,834 (85.6%) (85.0- 86.2 at 99% Confidence Interval) participants had abdominal obesity using Waist-to-Height Ratio, higher in women 12,397 (86.4%) than men 9,437 (84.5%). The mean age of the participants was 53.37±13.15 years and more than half 17,075 (55.7%) of all participants were female.

**Conclusions:**

The prevalence of abdominal obesity among hospital outpatients is higher than in other community-based studies. As hospitals attract large crowds and provide excellent opportunities for screening patients, their attendants, as well as providing opportunities for health promotion, we recommend the introduction of opportunistic obesity screening in all health facilities using weight to height ratio in a phased manner.

## INTRODUCTION

Abdominal obesity, abnormal or excessive fat accumulation that presents a risk to health, has become a global epidemic.^1^ The world is rapidly becoming obese.^2,3^ According to World Health Organization, the obesity rates have tripled since 1975. In 2016 more than 1.9 billion adults above 17 years were overweight and of those, over 650 million were obese.^4,5^ Nepal is following a similar trajectory^6-10^ as the country is rapidly undergoing urbanization, change in lifestyle, and dietary pattern.^6,11^

Based on the global burden of disease data, the cause of death from Non-communicable diseases (NCDs) in Nepal has reached 62% in 2017 from 36.1% in 2009.^12,13^ Measurement for Obesity among hospital outpatients is not a standard practice in most Low-and Middle-Income countries including Nepal.

This study aimed to find out the prevalence of abdominal obesity using the Waist-to-Height Ratio (WHtR) among outpatients in a tertiary level hospital.

## METHODS

This hospital-based descriptive cross-sectional study was conducted from January 2016 to December 2018 at Hospital for Children, Eye, ENT, and Rehabilitation Services (CHEERS), Bhaktapur, Nepal. Ethical approval was taken from the Ethical Review Board of Nepal Health Research Council (Reference no. 207/2019). Records of outpatients aged 35 years and above visiting the free Health Promotion and Screening service of CHEERS were reviewed. Pregnant women and people unable to stand properly were excluded from the analysis. Convenience sampling was done, and the sample size was calculated using the formula,

n = Z^2^ × (p × q) / e^2^

  = (2.576)^2^ × 0.5 × (1-0.5) / (0.01)^2^

  = 16,590

where,

n= required sample size
Z= 2.576, at 99% Confidence Interval (CI)
p= prevalence of abdominal obesity using the waist to height ratio among outpatients in a tertiary level hospital taken as 50% for maximum sample size
e= 1%, margin of error

The calculated sample size was 16,590. Adding a 20% non-response rate, the final calculated sample size was 20,737. However, records of 25,511 outpatients were included in the study.

Waist-to-Height Ratio^14^ (WHtR) 0.5 was considered as cut-off for abdominal obesity. Data entry was done in Excel (MS Office 2010). Data analysis was done in IBM Statistical Package for Social Sciences (version 24). Point estimate at 99% CI was calculated along with number and percentage. Continuous variables are shown as the mean±standard deviation (SD).

## RESULTS

Among 25,511 participants, 21,834 (85.6%) (85.0-86.2 at 99% Confidence Interval) participants had abdominal obesity. Abdominal Obesity was found higher in women 12,397 (86.4%) than men 9,437 (84.5%) (Table 1).

**Table 1:** Classification of Waist-to-Height Ratio (n = 25,511).

Category	Male	Female	Total
	n (%)	n (%)	n (%)
≥0.5	9,437 (84.5)	12,397 (86.4)	21,834 (85.6)
<0.5	1,733 (15.5)	1,944 (13.8)	3,677 (14.4)

The highest proportion of obesity was found in the age group 45-54 years, in both male 2,741 (87.3%) and female 3,414 (88.4%). The next highest group was participants from age group 55-64 years in both male 1,857 (86.1%) and female 2,320 (86.6). The prevalence of abdominal obesity gradually decreased as age increased (Table 2).

**Table 2:** The proportion of obesity according to age group (n = 25,511).

		≥ 0.5 n (%)	< 0.5	Total
Male	35-44	3,004 (83.9)	576 (16.1)	3580
	45-54	2,741 (87.3)	399 (12.7)	3140
	55-64	1,857 (86.1)	301 (13.9)	2158
	65-74	1,167 (82.4)	249 (17.6)	1416
	75 & above	668 (76.3)	208 (23.7)	876
Female	35-44	4,477 (88.0)	613 (12.9)	5090
	45-54	3,414 (88.4)	449 (11.6)	3863
	55-64	2,320 (86.6)	359 (13.4)	2679
	65-74	1,516 (81.7)	340 (13.4)	1856
	75 & above	670 (78.5)	183 (21.5)	853

Most of the participants were female 14,341 (56.2%). Regardless of gender, the highest number of participants were from the age group 35 to 44 years (32.1% male, 35.5% female) and their numbers gradually decreased as the age increased (Table 3).

**Table 3:** Age and gender distribution of participants (n = 25,511).

Age-group	Male n (%)	Female n (%)
35 to 44 years	3,580 (32.1)	5,090 (35.5)
45 to 54 years	3,140 (28.1)	3,863 (26.9)
55 to 64 years	2,158 (19.3)	2,679 (18.7)
65 to 74 years	1,416	1,856 (12.9)
75 & above years	876	853 (5.9)
Total	11,170	14,341

The mean (SD) age of male participants was 53.37±13.15 years and 52.41±12.77 years for female participants. The mean (SD) WC of males was 91.55±10.32cm and females were 87.56±11.03cm. Similarly, mean WHtR was 0.56±0.06, 0.58±0.07 for males and females respectively (Table 4).

**Table 4:** Distribution of mean and SD for different variables (n = 25,511).

Variables	Male	Female
	Mean±SD	Mean±SD
Age (years)	53.37±13.15	52.41±12.77
Height (cm)	163.61±7.44	151.54±6.75
Waist Circumference (CM)	91.55±10.32	87.56±11.03
Waist to Height Ratio	0.56 ± 0.06	0.58 ± 0.07

## DISCUSSION

This study analyzed the Waist-to-Height ratio of a large number of participants (25,511) visiting the free Health Promotion and Screening service of CHEERS in 3 year period. We choose Waist-to-Height ratio as the measure of abdominal obesity for this study as it has been shown to be a superior proxy than other obesity metrics for assessing the long-term risk of NCDs.^15-17^ There is an increasing body of evidence to support a trait of abdominal adipose deposition despite normal weight in South Asian populations.^18-21^ Besides the importance of abdominal obesity, very few studies measured abdominal obesity, almost all studies were community-based except a study done in India in outpatients^22^ which used Waist circumference and Waist-to-Hip ratio as obesity measurement and, none from Nepal hospitals.

In this study, the prevalence of abdominal obesity was found higher (85.6%) than the prevalence reported by another study done in South Korea (38.2%)^23^ Though this study was not comparable as the study was community-based. High prevalence in the present study may be due to differences in study design, a selection bias as people reporting to hospitals may have some or other conditions which may have had obesity at the background of their illness. However, the possibility of overestimation due to methodological error is very minimal because of the use of standardized instruments and trained personnel. The literature search revealed several reports of obesity based on inpatients but only one based on outpatient attendance.

This study found 86.3% prevalence of abdominal obesity among women and 84.5% among men. This finding is higher than the prevalence found in South Korea (male= 46.2% and female= 52.2%).^23^ The prevalence of abdominal obesity is also higher than other studies done in Slovak Republic (male=54.2%, female= 35.0%) and in India (51.9%).^24,25^ Higher abdominal obesity among women in this study may be because women (over 55.0%) have accumulated excess fat before 45 years of age. It is known that abdominal obesity may increase substantially with each pregnancy independent of total body fat^26^ which may explain higher obesity among women of childbearing age.

Recognizing the importance of abdominal obesity as a risk factor for many diseases conditions, we initiated this study because hospitals attract a large number of people such as patients, their escorts, and staff under one roof which allows screening of a huge number of people at little extra time and cost. Additionally, there is a captive population in the hospitals where service seekers are more amenable to the advice given by health workers. The other objective is to highlight how health systems are missing opportunities for diagnosis of obesity which has a profound impact on health.

As this is a hospital-based descriptive single-center study done using retrospective record review, the obesity estimation may be higher.

## CONCLUSIONS

The prevalence of obesity among hospital outpatients in our study is higher than other community-based studies, seriously signals the need for the health system leaders to formulate actions to detect and intervene before it develops into overt NCDs. Hospitals also provide great opportunities for health promotion to a receptive and concerned population. Therefore, we recommend to the Government of Nepal to introduce screening for obesity using WHtR at the first point of contact with the health system as well as during STEPS surveys as a superior metric and set up interventions in a phased manner to stem the rising tide of NCDs.
